# A case-control validation of Type D personality in Greek patients with stable coronary heart disease

**DOI:** 10.1186/1744-859X-12-38

**Published:** 2013-11-27

**Authors:** Christos Christodoulou, Athanasios Douzenis, Paula MC Mommersteeg, Loukianos Rallidis, Antonis Poulios, Vasiliki Efstathiou, Georgios Bouras, Christos Varounis, Panagiota Korkoliakou, John Palios, Dimitrios Th Kremastinos, Lefteris Lykouras

**Affiliations:** 1 Second Department of Psychiatry, "Attikon" General Hospital, University of Athens Medical School, Athens 12462, Greece; 2 CoRPS—Center of Research on Psychology in Somatic Diseases, Department of Medical and Clinical Psychology, Tilburg University, Tilburg 5000 LE, The Netherlands; 3 Second Department of Cardiology, "Attikon" General Hospital, University of Athens Medical School, Athens 12462, Greece

**Keywords:** Coronary heart disease, Type D personality, DS14, Validity, HADS

## Abstract

**Background:**

Type D personality has been associated with a variety of emotional and social difficulties as well as with poor prognosis in patients with established coronary heart disease (CHD). We examined the psychometric properties and validity of the Type D Scale-14 (DS14) and the prevalence of Type D personality among Greek patients with CHD while taking into account demographic; clinical, such as diabetes mellitus, hypertension, and hypercholesterolemia; as well as psychological variables such as depression, anxiety, and psychological stress.

**Methods:**

Ninety-six patients with stable coronary heart disease and 80 healthy participants from the general population completed the Greek version of the DS14 and the Hospital Anxiety and Depression Scale (HADS).

**Results:**

Cronbach's *α* coefficient for the negative affectivity (NA) and social inhibition (SI) subscales was 0.83 and 0.72 for the CHD and 0.88 and 0.76 for the control group, respectively. Internal-structural validity was assessed by a factor analysis (two-factor solution), and the factor structure of the original DS14 was replicated. Using the standardized cutoff point of NA ≥10 and SI ≥10, instead of the median scores, in order to have compatible results with the majority of studies, the prevalence of Type D personality was 51% for the CHD patients and 13% for the control group. Higher NA and SI were connected with higher anxiety, depression, and total psychological stress. Finally, more patients with CHD and Type D personality than those without were diagnosed with type 2 diabetes; however, no differences were observed in hypertension or hypercholesterolemia.

**Conclusions:**

These results indicate that the Type D construct is reliable and valid in a Greek population. The prevalence of Type D personality was higher in patients with stable coronary heart disease than in people from the general population. The DS14 subscales were positively correlated with higher anxiety, depression, and total psychological stress. Regarding other CHD risk factors, only diabetes mellitus was found more frequently in CHD patients with Type D personality.

## Background

Cardiac problems, such as coronary heart disease, myocardial infarction (MI), arrhythmias, and hypertension, are associated with acute and with chronic mental stress [1,2]. Depression and anxiety can be generally regarded as factors associated with increased morbidity and mortality of coronary heart disease [3].

However, studies suggest that the diagnosis itself is not the most decisive factor. Research has also indicated that what defines the depressed feeling, rather than the physical symptoms of depression, is important for coronary heart disease [4]. These symptoms reflect long-standing psychological attitudes, rather than a specific psychiatric condition. Several other negative emotions, apart from anxiety and depressed mood, have also been associated with coronary heart disease. These include anger, hostility, and physical exhaustion [5].

Personality is an important determinant of the emotional and physical health of patients with coronary heart disease [6]. Certain personality traits may promote while some others may hinder the development of social contacts and links, therefore strengthening or weakening a supportive social network, whose absence has been associated with increased morbidity and mortality in heart disease [4,7].

In recent years, studies have focused on negative emotions and social isolation as risk factors associated with the diagnosis and development of coronary heart disease, myocardial infarction, and cardiac death [8]. Thus, there is a research interest on Type D personality, also called 'distressed personality’. This type of personality is defined by two stable traits: negative affectivity (NA) which refers to a range of negative emotions experienced by the individual and social inhibition (SI) which refers to the expression of these negative emotions in social transactions leading to excessive distress. Type D personality has been associated with a variety of emotional and social difficulties as well as increased morbidity and mortality in patients with diagnosed heart disease [9-11]. Studies have focused on cardiac patients with myocardial infarction and coronary heart disease in general. In these studies, Type D personality was assessed as an independent risk factor for coronary heart disease [4,12,13]. Type D personality has been found to prospectively affect quality of life, poor response to treatment, and mortality in patients with myocardial infarction, chronic heart failure, hypertension, and peripheral arterial disease [9,14-16], though negative findings have been reported as well [17-19].

Taking into consideration the possible impact of personality traits on coronary heart disease, the validation of the scale and its correlation with other parameters would contribute to a more comprehensive control of the disease risk factors for Greek patients.

### Aim

The purposes of this study are (1) to assess the reliability and validity of the scale in a Greek population and (2) to assess the relationship between Type D personality and stable coronary heart disease, by taking into account demographic, clinical, and psychological data of the patients.

We hypothesize that people with Type D personality are more prevalent among patients with stable coronary heart disease compared to healthy controls in a Greek population and that this type of personality is related to clinical and psychopathological aspects.

## Methods

### Subjects

Stable coronary heart disease (CHD) patients who attended the outpatient cardiologic department of the "Attikon" University General Hospital of Athens, from January 2009 to February 2010, were asked to participate in this study, which is part of a wider research project of the cardiologic department regarding the management of dyslipidemia in patients with stable CHD [20]. Patients were included in the study if they had previously been hospitalized for acute coronary syndrome (ACS) more than 6 months ago, in order to be considered stable, or had undergone coronary artery bypass graft (CABG; >6 months) or coronary angiography for chest pain with documented CHD. Coronary artery stenosis was defined as ≥50% reduction in the lumen diameter of any of the three coronary arteries or their primary branches. Exclusion criteria were ACS or CABG within the previous 6 months, clinical evidence of heart failure (≥class II according to the New York Heart Association, chronic renal failure (creatinine levels >2 mg/dl (176.8 μmol/l)), and coexistent malignancy or inflammatory disease. During the study period (January 2009 to February 2010), cardiologists referred patients who fulfilled the aforementioned criteria. From a total of 152 patients, 39 were excluded due to insufficient knowledge of the Greek language, education level (less than 6 years, corresponding to elementary education in Greece; if there was, additionally, inadequate understanding of the questions), and various degrees of cognitive impairment. Seventeen patients refused to participate. The remaining 96 patients were included in the study. As a control group (CG), we used 80 healthy subjects from the general population matched for sex and age, who were recruited during the same time period. Subjects were not included if they suffered from cardiac diseases and hypertension, diabetes mellitus, hypercholesterolemia, or other life-threatening diseases; had various degrees of cognitive impairment; had a history of psychiatric disorders; or did not have sufficient knowledge of the Greek language in order to understand the questions. The participation of people in the control group was voluntary, without any financial compensation. The study protocol was approved by the Ethics Committee of the "Attikon" University General Hospital of Athens. All participants provided written informed consent, and the study was carried out in accordance with the Declaration of Helsinki.

### Procedure

For all subjects, demographic data (gender, age) and lifestyle factors, such as smoking and alcohol use, were reported. Clinical data from the patient's medical history, such as diabetes mellitus, hypertension, hypercholesterolemia, previous myocardial infarction, and medication history, were recorded. The following definitions were used: hypertension, blood pressure ≥140/90 mmHg and/or antihypertensive treatment; diabetes mellitus, fasting plasma glucose >125 mg/dl (6.9 mmol/l) and/or glucose-lowering treatment; and hypercholesterolemia, total cholesterol >200 mg/dl (5.2 mmol/l) and/or lipid-lowering treatment [20].

#### Type D personality

Two psychiatrists, bilingual in Greek and English, working independently translated the Type D Scale-14 (DS14) from the original English version [8] into Greek. Then they examined the two drafts and chose the expressions, item by item, that best conveyed the sense of the original. The new Greek text agreed was also given to another Greek psychiatrist, bilingual and fluent in English, who was requested to translate it back into English. The back-translated and original versions were found to be identical in terms of content on all items, with minor grammatical differences. The new Greek text was almost identical to the Greek version of the same scale as translated by Tziallas and colleagues [21]. It is worth noting that our study started prior to the publication of the aforementioned article including the validated scale in Greek.

All participants, both patients and controls, completed the Type D personality scale. The 14 items are rated on a 5-point Likert scale from 0 (false) to 4 (true). Seven items comprise NA which includes discomfort, anxiety (worry), and irritability, and seven items comprise the subscale SI which involves discomfort in social interactions, taciturnity-reticence, and lack of social poise. Individuals are classified as having Type D personality if both negative affectivity and social inhibition are rated greater than or equal to 10 [8]. The Greek version of the DS14 is presented in Additional file 1.

#### Hospital Anxiety and Depression Scale

The patients also completed the Hospital Anxiety and Depression Scale (HADS). This is a short self-assessment scale measuring anxiety and depression, which are the most common emotions that cause psychological distress in patients. The scale consists of two seven-item subscales that assess anxiety or depression excluding somatic symptoms. Responses to items are indicated on a 4-point Likert scale from 0 to 3 (score range 0–21), with a high score indicating more symptoms [22]. The scale has been translated and validated in Greek [23]. The total scores of depression and anxiety subscales were estimated separately, as well as their sum, as an assessment of psychological stress.

### Statistical analyses

Descriptive statistics were measured by calculating means or medians, standard deviations, minimum and maximum values, as well as absolute and relevant frequencies.

Normality was assessed by Kolmogorov-Smirnov's *Z* test. We then proceeded with two independent sample *t* tests. Despite the fact that the plots of both factors were skewed, we present the results of the aforementioned parametric test only; as we checked the comparisons' results with the non-parametric Mann–Whitney *U* test, they did not differ from those with the *t* test. Effect size was estimated with Cohen's *d* coefficient. Correlations were measured by Pearson's *r* correlation coefficient.

Concerning internal consistency, we calculated Cronbach's alpha coefficient and the corrected item-total correlation coefficients as well as the individual contribution of each item in the central tendency and variance.

The internal-structural validity of the scale was assessed by factor analysis (two-factor solution) in order to investigate whether the factor structure of the Greek version of the scale was the same as that of the prototype one.

The percentage ratio differences of the presence of Type D personality by the presence of CHD were assessed with the chi-square test.

Finally, a hierarchical multiple regression analysis was performed in order to estimate the prediction of HADS and its subscales (dependent variables), mainly from the two factors (NA and SI) of the DS14.

## Results

### Demographics and baseline characteristics

Ninety-six (mean age 59.03 ± 11.15, 89.6% males) patients with CHD and 80 (mean age 56.09 ± 9.94, 88.8% males) individuals from the general population as CG participated in the study. There were no differences in age or gender between the CHD and the control group (*n* = 176; age: *df* = 174, *t* = -1.86, *p* = 0.073; gender: *df* = 1, *χ*^2^ = 0.03, *p* = 0.859). Seventy-five (78%) of the patients with CHD and 64 (80%) of the CG were married (*df* = 1, *n* = 176, *χ*^2^ = 0.09, *p* = 0.79).

Concerning smoking, significantly more participants of the CG (41, 51.2%) than the CHD one (21, 21.9%) were smoking (*df* = 1, *n* = 176, *χ*^2^ = 15.37, *p* < 0.001). Similarly, more participants of the CG (27, 33.8%) than the CHD one (10, 10.4%) were using alcohol at the time of the study (*df* = 1, *n* = 176, *χ*^2^ = 14.31, *p* < 0.001). Ninety-five of the 96 patients were under treatment, with one or more of the following medications: antiplatelet treatment, lipid-lowering medication, beta-blockers, and angiotensin-converting enzyme inhibitors, at the time of examination.

### Type D validity

Concerning the NA factor's internal consistency, the overall sample's Cronbach alpha coefficient equalled 0.88. For the CG and CHD groups separately, the coefficients were 0.88 and 0.83, respectively. For the overall and CG samples, the corrected item-total correlation coefficients were moderate to high ranging from 0.56 and 0.61 for item 2 to 0.79 and 0.75 for item 13, respectively. For the CHD group, the correlation coefficients were rather low ranging from 0.27 to 0.60 for items 2 and 13, respectively. As far as the SI factor's internal consistency is concerned, the overall sample's Cronbach alpha coefficient equalled 0.77. For the CG and CHD groups separately, the coefficients were 0.76 and 0.72, respectively. For all the overall, CG, and CHD group samples, the corrected item-total correlation coefficients were low to moderate and even high for the CG ranging from 0.27, 0.18, and 0.21 for item 3 to 0.45, 0.73, and 0.57 for item 10, respectively.

The internal-structural validity of the two scales' factors was examined for both groups at the same time, through a factor analysis (Table 1). These two factors clearly reflected the negative affectivity and social inhibition subscales with satisfactory factor loadings ranging from 0.68 to 0.83 for the negative affectivity and 0.38 to 0.80 for social inhibition. NA and SI were positively correlated according to Pearson's *r* correlation (*r =* 0.41, *p* < 0.001).

**Table 1:** Two-factor solution's rotated factor loadings, eigenvalues, and variance explained

**Item**	**Factor I**	**Factor II**
Negative affectivity^a^		
(13) I am often down in the dumps	**0.83**	0.22
(5) I am often irritated	**0.81**	0.08
(12) I often find myself worrying about something	**0.78**	0.01
(4) I often feel unhappy	**0.78**	0.18
(7) I take a gloomy view of things	**0.71**	0.28
(9) I am often in a bad mood	**0.69**	0.39
(2) I often make a fuss about unimportant things	**0.68**	0.02
Social inhibition^b^		
(10) I am a closed kind person	0.11	**0.80**
(8) I find it hard to start a conversation	0.16	**0.68**
(6) I often feel inhibited in social interactions	0.26	**0.67**
(14) When socializing, I don't find the right thing to talk about	0.24	**0.66**
(11) I would rather keep other people at a distance	0.20	**0.63**
(1) I make contact easily when I meet people^c^	-0.03	**0.62**
(3) I often talk to strangers^c^	0.22	**0.38**

^a^Chronbach's alpha = 0.88, eigenvalue I = 4.19, variance explained = 29.94%; ^b^Chronbach's alpha = 0.77, eigenvalue II = 1.66, variance explained = 23.02%; ^c^reverse-keyed.

### Type D prevalence

The median scores of the two factors were 10 for NA and 9 for SI. However, we used the standardized cutoff point ≥10 for both factors in order to discriminate the participants as Type D personalities with a view to having compatible results with the majority of studies which use this cutoff point instead of the medians.

There were significant differences in the presence of Type D personality between the CHD (51%, *n* = 49) and the control (12.5%, *n* = 10, *χ*^2^ (*df* = 1, *n* = 176) = 29.09, *p* < 0.001) group.

In addition, the control group reported less negative affectivity (*M* = 6.15, SD = 5.71) as compared to the CHD group (*M* = 13.71, SD = 6.41) (*t* (174) = -8.18, *p* < 0.001). Similarly, the control group reported less social inhibition (*M* = 7.45, SD = 4.57) in comparison to the CHD group (*M* = 11.82, SD = 5.68) (*t* (174) = -5.66, *p* < 0.001).

**Figure 1:**
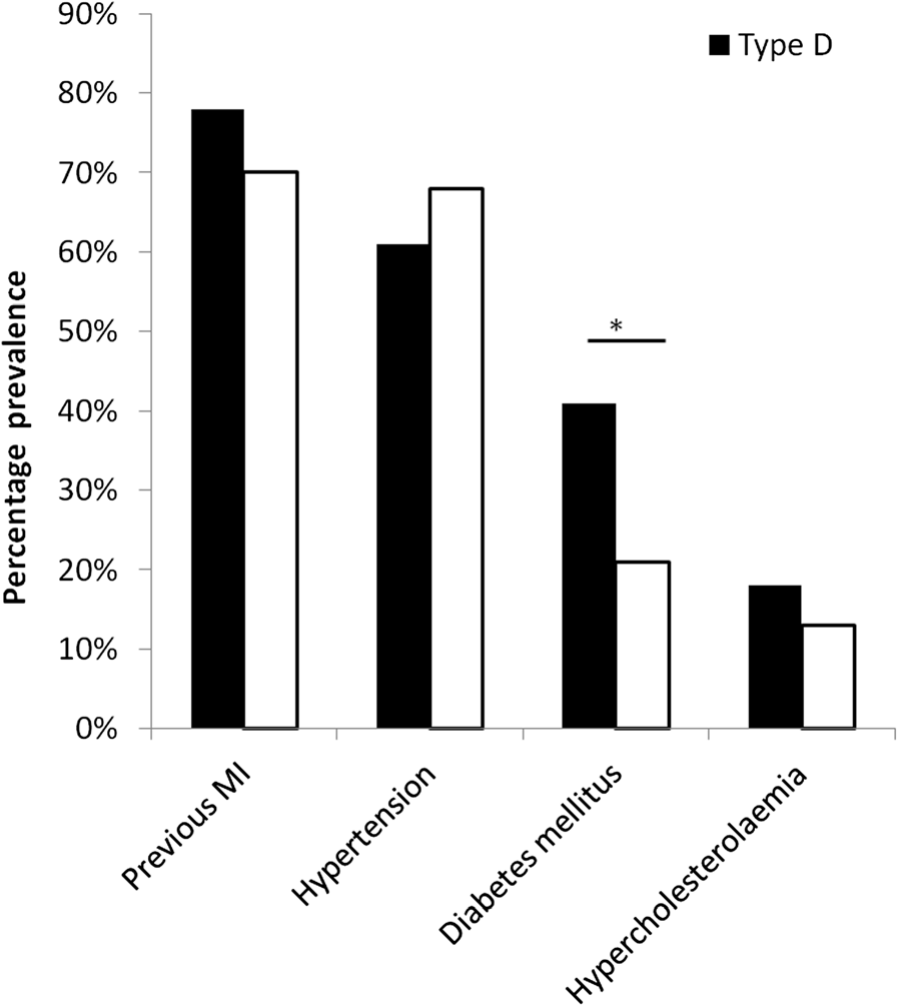
**Type D stratified comorbidity in the coronary heart disease patient group (*n*** **= 96).** *p < 0.05.

Within the CHD group, the people with Type D personality showed a significant increased prevalence of having type 2 diabetes (41% versus 21%, *χ*^2^ (*df* = 1, *n* = 96) = 4.26, *p* = 0.039) compared to the non-Type D patients (Figure 1). There was no difference in previous MI (78% versus 70%, *χ*^2^ (*df* = 1, *n* = 96) = 0.67, *p* = 0.412), hypertension (61% versus 68%, *χ*^2^ (*df* = 1, *n* = 96) = 0.49, *p* = 0.482), or hypercholesterolemia (18% versus 13%, *χ*^2^ (*df* = 1, *n* = 96) = 0.57, *p* = 0.44) between the groups (Figure 1).

### Hospital Anxiety and Depression Scale

The Pearson *r* coefficient was also used to assess the correlation between depression, anxiety, and psychological stress (total score of HADS) for the cardiovascular patients. All coefficients were statistically significant (*p* < 0.001) and of positive direction, particularly anxiety, 0.76 with NA and 0.33 with SI; depression, 0.63 with NA and 0.43 with SI; and total psychological stress, 0.77 with NA and 0.41 with SI. In all cases, higher NA and SI are connected with higher anxiety, depression, and total psychological stress.

We then proceeded by conducting three hierarchical multiple regression analyses in order to estimate the prediction of anxiety, depression, and total psychological stress (dependent variables) from age and sex (first step), NA and SI (second step), and all the possible predictors' interactions (third step) whose contribution was in all cases not significant and thus not presented. Concerning the prediction of anxiety, depression, and psychological stress in patients with CHD, the results of the analysis are presented on Table 2.

**Table 2:** Hierarchical multiple regression analysis for anxiety, depression, and psychological stress in patients with CHD

**Predictors**	**Anxiety coefficient**		**Depression coefficient**		**Psychological stress coefficient**	
	***β***	***t***	***β***	***t***	***β***	***t***
Gender	0.34	2.46***	0.14	1.41	0.27	2.74**
Age	-0.08	-0.87	0.13	1.31	0.02	0.18
Negative affectivity	0.69	10.22***	0.58	7.41***	0.70	10.89***
Social inhibition	0.16	2.31*	0.30	3.82***	0.24	3.77***

Dependent variables: anxiety (method enter): *R*^2^ *=* 0.61, *F*(4.90) = 37.6, *p* < 0.01; depression (method enter): *R*^2^ = 0.48, *F*(4.90) = 22.32, *p* < 0.001; psychological stress (method enter): *R*^2^ = 0.64, *F*(4.90) = 43.07, *p* < 0.001. *p < 0.05, **p < 0.01, ***p < 0.001.

According to the beta coefficients, only gender contributes significantly to the prediction, with women having higher levels of anxiety (it is noteworthy that the number of females in our sample is small). Finally, according to the beta coefficients, higher levels of both NA and SI are associated with higher levels of anxiety, depression, and psychological stress (Table 2).

## Discussion

The aim of this study was to examine the reliability and validity of the DS14 in patients with stable coronary heart disease. The relationship between Type D personality and stable coronary heart disease based on demographic, clinical, and psychological factors was also examined. The validity of the scale was found sufficient, and the prevalence of Type D personality was higher in patients with stable coronary heart disease than in people from the general population. Higher NA and SI were positively correlated with higher levels of anxiety, depression, and psychological stress. Regarding other coronary heart disease risk factors, only type 2 diabetes was found to be more prevalent in patients with CHD.

The Greek version of the DS14 demonstrated good psychometric properties and a sufficient degree of internal consistency. Factor analysis indicated a two-factor solution. These were labeled as the NA and SI factors in accordance with their corresponding high-loading items. These finding are in accordance with previous studies [7,8].

The median scores of the two factors were 10 for NA and 9 for SI. Grande and colleagues [24] suggested medians of 10 and 9 for the NA and SI subscales, respectively, as well. However, we used cutoff point ≥10 for both subscales in order to have compatible results with the majority of studies which use this standardized cutoff point instead of the median scores [8].

Using the recommended aforementioned cutoff point to define Type D personality, 51% of the cardiovascular patients, 12.5% of the control group, and 33.5% of the total sample were categorized as having Type D personality. The prevalence of 51% in the cardiac sample of our study was higher than those found in other European and Asian samples. In European countries, the prevalence of Type D personality among patients with coronary heart disease ranged from 15% to 36% [25,26], and in Asian countries (China and Korea), it ranged from 26.1% to 31.4% [27-29]. The current study showed that the prevalence of Type D personality in the control group was 12.5%. In previous research conducted in Europe, the prevalence of the scale in the general population or healthy controls was 13.3% to 38.5% [30,31].

Denollet [8] found that Type D personality was more prevalent in hypertension patients compared with individuals from the general population (53% versus 19%), whereas we did not observe a difference in Type D personality prevalence in the patients with comorbid hypertension.

Also, in Italy, the prevalence of Type D personality was estimated up to 39% in patients attending a program of cardiac rehabilitation [32]. Moreover, in a recent Greek study, the prevalence of Type D personality for patients who met the diagnostic criteria for the metabolic syndrome was 44%, a percentage significantly higher than that of the control group (15%) [21].

Finally, according to a recent review article, it seems that Type D personality among patients with ischemic heart disease is more prevalent in southern (37%) as well as eastern (35%) European countries, when compared to western (27%) and northern (24%) European countries [33]. The above results indicate that the prevalence of Type D personality in patients with CHD varies in different countries. The reason for this variation remains unclear. Some studies report that ethnic and cultural differences among countries may be responsible for variation in Type D personality prevalence [27,29,33]. Depression and other personality traits seem to differentiate across country clusters, with the southern and eastern European ones displaying higher prevalence rates [33].

In our study, there is a large difference between the prevalence of personality Type D in the control group and cardiac patients. The majority of Greek people, as well as people from other Mediterranean countries, have been described as being both socially and temperamentally more extroverted and also more optimistic about the future. These could justify the low score of those from the general population. On the other hand, in these countries, people with inhibited sociality and negative emotions could be characterized, culturally, as having abnormal features (traits). These individuals have traits that are in contrast to the majority of people around them, and this may contribute to a higher rating than average and, possibly, to an increased vulnerability to a cardiac event.

It should be noted that the study was conducted prior to the onset of the economic crisis in Greece, which gradually started from April 2010.

According to our findings, higher NA and SI are associated with higher anxiety, depression, and total psychological stress. Moreover, higher levels of both NA and SI were correlated with higher levels of anxiety, depression, and psychological stress. In a study [25], univariable linear and logistic regression analyses were used in order to confirm that Type D personality was associated with an increased risk of anxiety and depression as measured by HADS. In the same study, multivariable linear regression showed that Type D personality remained independently associated with an increased risk of anxiety and depression adjusting for baseline sociodemographic and clinical characteristics [25]. Furthermore, another recent study using the same scale (HADS) found high correlation between negative affectivity and anxiety and depression [34].

According to our results, more patients with CHD and Type D personality than those without Type D personality were diagnosed with diabetes mellitus, but no differences were observed in hypertension or hypercholesterolemia. Mommersteeg and colleagues [35] did not find significant differences in diabetes mellitus, but found differences in two other components of the metabolic syndrome (lipid abnormality and hypertension), between people with Type D and non-Type D personalities. Similarly, Svansdottir and colleagues [36] found that Type D personality was associated with high prevalence of hypertension, diabetes, blood lipids, wider waist circumference, and elevated body mass index. Nevertheless, several studies did not find differences regarding diabetes mellitus or other usual cardiovascular risk factors among patients with Type D and non-Type D personalities [25,28]. Overall, the findings of the studies are inconsistent, and further research is needed to establish an accurate association between the type of personality and other risk factors for CHD.

Some study limitations should be mentioned. The findings of the present study should be interpreted with some caution. The total sample size is not large, and the number of women is particularly low. Thereby, examining the findings separately for sex was not valid. Test-retest reliability could not be assessed in order to confirm the temporal stability of the scale.

## Conclusions

In conclusion, the results of the current study in a Greek sample of cardiovascular patients and the general population confirm that Type D is a valid and reliable personality construct, with good psychometric properties and which is compatible with other measures associated with Type D personality. The prevalence of Type D personality was higher in patients with stable coronary heart disease than people from the general population. The subscales of the DS14 were positively correlated with higher levels of anxiety, depression, and psychological stress. Apart from diabetes mellitus, no other coronary heart disease risk factors were found more frequently in CHD patients with Type D personality. Further research is needed to examine whether Type D personality is an independent variable from other risk factors for cardiovascular morbidity and/or mortality in Greek patients.

## Abbreviations

ACS: Acute coronary syndrome; CABG: Coronary artery bypass graft; CG: Control group; CHD: Coronary heart disease; DS14: Type D Scale-14; HADS: Hospital Anxiety and Depression Scale; MI: Myocardial infarction; NA: Negative affectivity; SI: Social inhibition.

## Competing interests

The authors declare that they have no competing interests.

## Authors' contributions

CC was a co-designer of the study, wrote the manuscript, and corrected the final draft. AD was a co-designer of the study and revised and made critical corrections in the manuscript. PMCM participated in the data interpretation and revised and made critical and substantial corrections in the manuscript. LR was a co-designer of the study and participated in the data collection. AP did the statistical analysis and interpretation of the data. VE participated in drafting and reviewing the manuscript. GB, CV, PK, and JP participated in the data collection and processing. DThK was a co-designer of the study. LL was a co-designer of the study and gave final approval to the published version. All authors read and approved the final manuscript.

## Supplementary Material

Additional file 1 **Greek version of Type D Scale-14 (DS14).** Additional file 1 contains the Greek version of the DS14. Its scoring as well as its assessment is in accordance to the original version of the scale. Click here for file
