# Supplementary material for: A case-control validation of Type D personality in Greek patients with stable coronary heart disease
Source: Ann Gen Psychiatry. 2013 Nov 27;12:38. doi: 10.1186/1744-859X-12-38 (PMC4175478; doi:10.1186/1744-859X-12-38)
Supplement: Additional file 1 — Greek version of Type D Scale-14 (DS14). Additional file 1 contains the Greek version of the DS14. Its scoring as well as its assessment is in accordance to the original version of the scale. [file 1744-859X-12-38-S1.doc]

**Additional file 1: Greek version of Type D Scale-14** (DS14)

| Παρακάτω υπάρχουν κάποιες φράσεις με τις οποίες οι άνθρωποι συχνά περιγράφουν τον εαυτό τους. Διαβάστε κάθε φράση και βάλτε σε κύκλο τον σωστό αριθμό για να απαντήσετε. Δεν υπάρχουν σωστές και λάθος απαντήσεις, το μόνο που έχει σημασία είναι η εντύπωση που σχηματίζετε γενικά. | | | | | |
| --- | --- | --- | --- | --- | --- |
| **0=Λάθος 1= Μάλλον λάθος 2=Ουδέτερο 3=Μάλλον σωστό 4=Σωστό** | | | | | |
| 1. Κάνω εύκολα επαφή όταν συναντώ ανθρώπους………………… | 0 | 1 | 2 | 3 | 4 |
| 2. Συχνά κάνω φασαρία για πράγματα που δεν έχουν σημασία……. | 0 | 1 | 2 | 3 | 4 |
| 3. Μιλώ συχνά σε αγνώστους………………………………………. | 0 | 1 | 2 | 3 | 4 |
| 4.Συχνά αισθάνομαι δυστυχισμένος………………………………… | 0 | 1 | 2 | 3 | 4 |
| 5. Εκνευρίζομαι συχνά……………………………………………… | 0 | 1 | 2 | 3 | 4 |
| 6. Συχνά αισθάνομαι άβολα στις κοινωνικές συναναστροφές….. | 0 | 1 | 2 | 3 | 4 |
| 7. Βλέπω μαύρα τα πράγματα………………………………………… | 0 | 1 | 2 | 3 | 4 |
| 8. Δυσκολεύομαι να ξεκινήσω μια συζήτηση………………………… | 0 | 1 | 2 | 3 | 4 |
| 9. Συχνά έχω κακή διάθεση………………………………………….. | 0 | 1 | 2 | 3 | 4 |
| 10. Είμαι κλειστός τύπος…………………………………………….. | 0 | 1 | 2 | 3 | 4 |
| 11. Θα προτιμούσα να κρατώ τους άλλους σε απόσταση…………….. | 0 | 1 | 2 | 3 | 4 |
| 12. Συχνά ανησυχώ για κάτι………………………………………... | 0 | 1 | 2 | 3 | 4 |
| 13. Έχω συχνά τις μαύρες μου……………………………………… | 0 | 1 | 2 | 3 | 4 |
| 14. Στις κοινωνικές συναναστροφές δεν βρίσκω τα κατάλληλα  θέματα να συζητήσω | 0 | 1 | 2 | 3 | 4 |

Negative Affectivity = sum of scores on items 2+4+5+7+9+12+13.

Social Inhibition = sum of scores on items 1[reversed]+3[reversed]+6+8+10+11+14.
